# The burden of headache is associated to pain interference, depression and headache duration in chronic tension type headache: a 1-year longitudinal study

**DOI:** 10.1186/s10194-017-0829-8

**Published:** 2017-12-28

**Authors:** Stella Fuensalida-Novo, Maria Palacios-Ceña, Juan J. Fernández-Muñoz, Matteo Castaldo, Kelun Wang, Antonella Catena, Lars Arendt-Nielsen, César Fernández-de-las-Peñas

**Affiliations:** 1 Department Physical Therapy, Occupational Therapy, Rehabilitation, and Physical Medicine, Facultad de Ciencias de la Salud, University Rey Juan Carlos, Avenida de Atenas s/n, 28922 Alcorcón, Madrid, Spain; 2 Center for Sensory-Motor Interaction (SMI), Department of Health Science and Technology, Faculty of Medicine, Aalborg University, Aalborg, Denmark; 3 Department of Psychology, Universidad Rey Juan Carlos, Alcorcón, Spain; 4 Master in Sport Physiotherapy, University of Siena, Siena, Italy; 5 Poliambulatorio Fisiocenter, Collecchio, (Parma), Italy

**Keywords:** Tension type headache, Burden, Depression, Emotional, Pain

## Abstract

**Background:**

To investigate variables associated at one year (longitudinal design) with the physical or emotional component of burden in chronic tension type headache (CTTH).

**Methods:**

One hundred and thirty (*n* = 130) individuals with CTTH participated in this longitudinal study. Clinical features were collected with a 4-weeks headache diary at baseline and 1-year follow-up. The burden of headache was assessed at baseline and one -year follow-up with the Headache Disability Inventory (HDI), physical (HDI-P) or emotional (HDI-E) component. Sleep quality (Pittsburgh Sleep Quality Index), anxiety and depression (Hospital Anxiety and Depression Scale-HADS), and quality of life (SF-36) were also assessed at baseline. Hierarchical regression analyses were conducted to determine the associations between the baseline variables and the headache burden at 1-year. Simple mediation models were also applied to determine the potential mediation effect of any intermediary variable.

**Results:**

Regression analyses revealed that baseline pain interference and depression explained 32% of the variance in the emotional burden of headache, whereas baseline emotional burden of the headache, pain interference, and headache duration explained 51% of the variance in the physical burden of headache (*P* < .01) at 1-year. The mediation models observed that the effect of baseline pain interference on emotional burden of headache at 1-year was mediated through baseline depression, whereas the effect of baseline pain interference on the physical burden of headache at 1-year was mediated through baseline emotional burden of headache (both *P* < .05).

**Conclusions:**

The current study found a longitudinal interaction between pain interference and depression with the burden of headache in individuals with CTTH.

## Background

Tension type headache is a common headache disorder showing a global prevalence of 42% in the general population [1] and an important socio-economic impact [2]. In the last Global Burden of Disease Study, headache was found to be the 3rd most prevalent pain condition in terms of global prevalence, the 6th in terms of global incidence and 28th in terms of years of life lived with disability [3]. The Eurolight project found that the burden of migraine and TTH in Europe is substantial, mostly related to lost days from work, lost days with household activity, lost family, social or leisure activities [4]. The general costs of headache, most related to migraine and TTH, in Europe in 2010 were €13.8 billion [5]. The Eurolight project estimated that indirect costs accounted for 92% of the financial burden of TTH [6]. In fact, recent data derived from the Eurolight project have confirmed that the burden of TTH is also substantial in middle-income European countries such as Lithuania [7]. Similarly, headache burden is also similar in low-income countries, e.g., Ethiopia [8]. Therefore a better understanding of those potential variables associated with the burden of headache can assist clinicians in understanding which factors may play an important role in the management of TTH.

The concept of burden can be defined from different viewpoints. The Eurolight project focused, but not limited, the definition of burden in relation to work productivity [4, 6], although this project clearly shows that headache has negative impact on different aspects of life including education, career and earnings, family, or social life. Therefore, the term burden includes several components (physical or emotional) of an individual. Lampl et al. defined the term burden as “any loss of health or well-being attributable to a headache disorder” [9]. This definition of burden will be used in the current study.

It seems clear that headache can have physical and emotional repercussions on the life of the individual. Therefore, emotional factors may be crucial for the burden and perception of the disease. There is evidence suggesting that subjects with TTH exhibit co-morbid anxiety, depression [10], or sleep disturbances [11]. Some studies have previously investigated the association between depression and the burden of headache, but most of them included patients with migraine, but not TTH [12, 13]. Zebenholzer et al. observed that coexistence of depression and anxiety had a significant impact on the burden in patients with TTH and migraine [14]. It would be conceivable that different variables can interact at different levels to headache-associated burden. No study has previously investigated variables associated with the burden of headache in patients with TTH in a longitudinal design. Therefore, the purpose of the current study was to investigate potential variables associated at one year (longitudinal design) with the physical or emotional component of burden in a cohort of patients with chronic tension type headache (CTTH).

## Methods

### Study design

The current analysis is included as part of a multicenter international headache study. Some patients from the current study were also included in a previous part of the study which data have been already previously published [15]. This study presents new data by including new patients, different outcomes, different statistical analysis, and a different design since we had used a longitudinal desing, instead just only a cross-sectional design.

### Participants

Patients with a diagnosis of TTH were recruited from three different university-based hospitals (Hospital Universitario Fundación Alcorcón-Spain, Aalborg University Hospital-Denmark, University Hospitals Parma Medical Center-Italy) from September 2014 to June 2016. Diagnosis was conducted according to the criteria of the International Classification of Headache Disorders, third edition (ICHD3 beta, 2013) down to third-digit level (codes 2.2, 2.3) by a neurologist expert in headaches [16]. They were excluded if presented: 1, epidosic headaches; 2, other primary or secondary headaches; 3, medication overuse headache as defined by the ICHD-III; 4, history of neck or head trauma; 5, any systemic degenerative disease; 6, diagnosis of fibromyalgia syndrome; 7, have received anesthetic blocks or botulinum toxin the previous 6 months; 8, have received physical treatment in the neck or head the previous 6 months; or, 8, pregnancy.

All participants read and signed a consent form prior to their participation. The local Ethics Committee approved the study (URJC 23/2014, HUFA 14/104, Aalborg N20140063, CESU 5/2015).

### Headache diary

A headache diary for 4 weeks was used to substantiate the diagnosis and to record the headache clinical features [17]. This diary was recorded at baseline and at one-year follow-up. On the diary, patients registered the frequency of headaches (days per week), the headache intensity on an 11points numerical pain rate scale [18] (NPRS; 0: no pain, 10: the maximum pain), and the duration of each headache attack (hours per day).

### Burden of headache

The Headache Disability Inventory (HDI) was used to assess the burden of headache. This questionnaire uses 25 items that inquire about the perceived impact of headache on emotional functioning and daily life activities [19]. Possible answers for each item include YES (4 points), SOMETIMES (2 points) and NO (0 points). Thirteen items assess the emotional burden (HDI-E, maximum score: 52) whereas the remaining 12 items assess the physical burden (HDI-P, maximum score: 48). A greater score suggests a greater burden of headache for each subscale. The HDI has exhibited good stability at short and long-term in patients with headache [20]. The HDI was assessed at baseline and at 1-year follow-up. The main outcome of this study was the burden of headache (HDI) at one-year follow-up.

### Anxiety and depressive symptoms

The Hospital Anxiety and Depression Scale (HADS) is a 14-items self-report screening scale indicating the presence of anxiety and depressive symptom [21]. It consists of 7 items for evaluating anxiety (HADS-A) and 7 for depression (HADS-D). Each item scores on a Likert scale (0–3) giving a maximum score of 21 points for each subscale [22]. The HADS has shown good validity and internal consistency for being used in subjects with headache [23]. Anxiety and depressive symptoms were assessed at baseline.

### Sleep quality

The Pittsburgh Sleep Quality Index (PSQI) as used to assess sleep quality [24]. This questionnaire assesses the quality of sleep over the previous month by including 19 self-rated questions and 5 questions answered by bedmates or roommates. Items use varying response categories recording usual bed time, usual wake time, number of actual hours slept, and number of minutes to fall asleep. All questions are answered on a Likert-type scale (0–3). The total score ranges from 0 to 21 where higher score indicates worse sleep quality. This questionnaire has good internal consistency and test-retest reliability [25, 26]. Sleep quality was assessed at baseline.

### Health-related quality of life

Quality of life was assessed with the Medical Outcomes Study Short Form 36 (SF-36) questionnaire [27]. This questionnaire includes the following 8 domains: physical functioning, physical role, bodily pain (pain interference), general health, vitality, social function, role-emotional, and mental health. Total score range from 0 (the lowest quality of life) to 100 (the highest quality of life) [28]. Health-related quality of life was assessed at baseline.

### Statistical analysis

Means and confidence intervals were calculated to describe the outcomes. The Kolmogorov-Smirnov test revealed that all data had a normal distribution (*P* > .05). To determine the relationship between the dependent measure (the emotional or physical burden of headache at one-year follow-up) and the independent outcomes (headache intensity, headache duration, headache frequency, sleep quality, HADS-D, HADS-A, physical functioning, physical role, pain interference, general health, vitality, social function, role-emotional, mental health at baseline), several Pearson product-moment correlation coefficients were first assessed. This correlational statistical analysis was used to check for multicollinearity and shared variance between the outcomes.

First, two regression models were used to assess the independent variables that contributed significantly to the variance in the emotional (HDI-E) and physical (HDI-P) burden of headache, separately. To examine the proportions of explained variance of the burden of headache, a hierarchical regression analysis was conducted. Changes in *R* ^2^ were reported after each step of the regression model to determine the association of the additional variables. Last, variables that significantly contributed to the score on the emotional or physical burden of headache were selected for inclusion into parsimonious final regression model. The significance criterion of the critical F value for entry into the regression equation was set at *P* < .05.

After the stepwise regression analyses, simple mediation models were applied to determine the potential mediation effect of depression (HADS-D) or emotional burden of headache (HDI-E) in the correlation between pain interference (bodily pain) and the emotional (HDI-E) or physical (HDI-P) burden of headache at 12 months, respectively. According to Baron and Kelly [29] in order to check a mediation hypothesis, previously it is necessary to develop several simple regression models between all variables included at the model for checking the following steps: 1, pain interference (bodily pain) as the predictable variable has a significant correlation with the emotional burden of headache (HDI-E) at one-year follow-up; 2, the predictive variable is related significantly with depression (HADS-D); 3, checking the mediated variable, in the first case, depression (HADS-D), is related with the criteria variable (the emotional burden of headache at 1-year) when the effect from the predictable variable is constant; and, 4, showing as the direct effect from pain interference (bodily pain) is significantly lower than when the mediated variable is included at the model (indirect effect) [30].

## Results

### Clinical data of the sample

A total of 200 individuals with headache were screened for possible eligibility criteria. Finally, 172 patients with CTTH (120 women, 50 men, mean age: 48 ± 15 years) satisfied all eligibility criteria, agreed to participate and signed the informed consent at baseline. Twenty-eight were excluded for the following reasons: co-morbid migraine (*n* = 17), episodic tension type headache (*n* = 5) previous whiplash (*n* = 3), fibromyalgia (*n* = 2) and medication overuse headache (n = 1). One hundred and thirty (*n* = 130, 76%, 95 women, 35 men, mean age: 47 ± 20 years) were also assessed at one-year follow-up and therefore included in the main analysis.

### Correlation analysis

Pearson’s correlation coefficients and the descriptive analysis between outcome variables are summarized in Table 1. Significant positive correlations were observed between the emotional burden of headache (HDI-E) at one-year and headache frequency (*r* = .281; *P* = .015), sleep quality (*r* = .326; *P* = .004), and depression (*r* = .408; *P* < .001) at baseline: the higher the frequency of the headaches, the worse the sleep quality, or the higher the depressive symptoms at baseline, the higher the emotional burden of the headache one year after. Significant negative correlations between the emotional burden of headache (HDI-E) at 1-year and pain interference (*r* = −.508; *P* < .001), vitality (*r* = −.374; *P* = .001) or mental health (*r* = −.343; *P* = .002) at baseline were also found: the lower the vitality, pain interference or mental health score, i.e., the lower vitality, the higher experience of pain or the worse mental health, at baseline, the higher the emotional burden of the headache one year after.

**Table 1:** Pearson-Product Moment Correlation Matrix for Functional and Psychological Variables at Baseline Statistically Associated with the Physical or Emotional Burden of Headache at One Year

	Mean	95% CI	1	2	3	4	5	6	7	8	9
1. HDI-E at 12 months (0–52)	16.1	13.1–19.1									
2. HDI-P at 12 months (0–48)	20.5	17.7–23.3	.783**								
3. Headache Intensity (0–10)	5.7	5.4–6.0	n.s	228*							
4. Headache Duration (hours/day)	7.7	6.9–8.5	n.s	.376**	.335**						
5. Headache Frequency (days/month)	19.1	17.3–20.9	.281*	.306**	.247*	.245*					
6. Pittsburg Questionnaire (0–21)	8.8	7.9–9.7	.326**	.291*	.284**	n.s	.193*				
7. HADS-D (0–21)	9.5	8.6–10.4	.408**	.330**	.199*	n.s	.209*	.451**			
8. Bodily Pain (SF-36, 0–100)	47.3	42.3–52.3	−.508**	−.556**	−.190**	n.s	−.222**	−.362**	−.447**		
9. Vitality (SF-36, 0–100)	46.3	41.6–51.0	−.374**	−.453**	n.s	n.s	n.s	−.418**	−.615**	.608**	
10. Mental Health (SF-36, 0–100)	51.1	46.4–55.8	−.343**	−.254*	−.206*	n.s	n.s	−.386**	−.730**	.447**	.647**

*95% CI* 95% confidence interval, *HDI* Headache Disability Inventory (*E* Emotional, *P* Physical), *HADS* Hospital Anxiety and Depression Scale (*D* Depression)

*P < 0.05; **P < 0.01

Significant positive correlations were observed between the physical burden of headache (HDI-P) at 1-year and headache intensity (*r* = .228; *P* = .045), frequency (*r* = .306; *P* = .008) and duration (*r* = .376; *P* = .009), sleep quality (*r* = .291; *P* = .01), and depression (*r* = .330; *P* = .004) at baseline: the higher the headache frequency, the higher the intensity of headache, the longer the headache duration, the worse the sleep quality, or the higher the depressive symptoms at baseline, the higher the physical burden of the headache one year after. Finally, significant negative correlations between the physical burden of the headache (HDI-P) at one-year and pain interference (*r* = −.556; *P* < .001), vitality (*r* = −.453; P < .001) or mental health (*r* = −.254; *P* = .03) at baseline were also observed: the lower the vitality, the pain interference or mental health score, i.e., the lower vitality, the higher experience of pain or the worse mental health at baseline, the higher the physical burden of the headache one year after.

### Regression analyses

Table 2 summarizes the hierarchical regression analysis conducted for the emotional burden of headache (HDI-E) at one-year. In this analysis, baseline pain interference (bodily pain) approximately contributed 27.2% (*P* < .001), whereas baseline depression (HADS-D) contributed an additional 5% (P < .001) to the variance of emotional burden of headache (HDI-E) at one-year follow-up. When combined, both variables explained 32.2% of the variance in the emotional burden of headache (r^2^ adjusted: 0.322, F = 11.33, *P* < .01).

**Table 2:** Summary of Stepwise Regression Analyses to Determine Baseline Predictors of the Emotional Burden of Headache at One Year (r^2^ = 32.2%)

Independent Variable	*B*	SE B	β	t	F	P
Step 1						
Bodily Pain	−.272	.052	−.532	−5.246	27.51	<0.001
Step 2						
Bodily Pain	−.222	.057	−.433	−3.925	11.33	<0.01
HADS-D	.689	.342	.222	2.014		

R^2^ = .272 for step 1; R^2^ = .322 for step 2

*HADS* Hospital Anxiety and Depression Scale (*D* Depression)

The hierarchical regression analysis conducted for the physical burden of headache (HDI-P) at one-year is summarized in Table 3. In this analysis, baseline emotional burden of headache (HDI-E) contributed 46% (*P* < .001), pain interference (bodily pain) an additional 6% (*P* < .01) and baseline headache duration an additional 3% (*P* < .001) of the variance of physical burden of headache (HDI-P) at one-year. When combined, all variables explained 51.1% of the variance in the physical burden of headache (r^2^ adjusted: 0.511, F = 27.77, *P* < .01).

**Table 3:** Summary of Stepwise Regression Analyses to Determine Baseline Predictors of the Physical Burden of Headache at One Year (r^2^ = 55.1%)

Independent Variable	*B*	SE B	Β	t	F	P
Step 1						
HDI-E	.598	.077	.678	7.721	59.61	<0.001
Step 2						
HDI-E	.453	.088	.514	5.143	37.54	<0.01
Bodily Pain	−.144	.048	−.297	−2.790		
Step 3						
HDI-E	.423	.087	.480	4.852	27.77	<0.01
Bodily Pain	−.148	.047	−.305	−3.124		
Headache Duration	.513	.243	.174	2.113		

R^2^ = .460 for step 1 R^2^ = .521 for step 2; R^2^ = .551 for step 3

*HDI* Headache Disability Inventory (*E* Emotional)

### Mediation effects

**Fig. 1:**
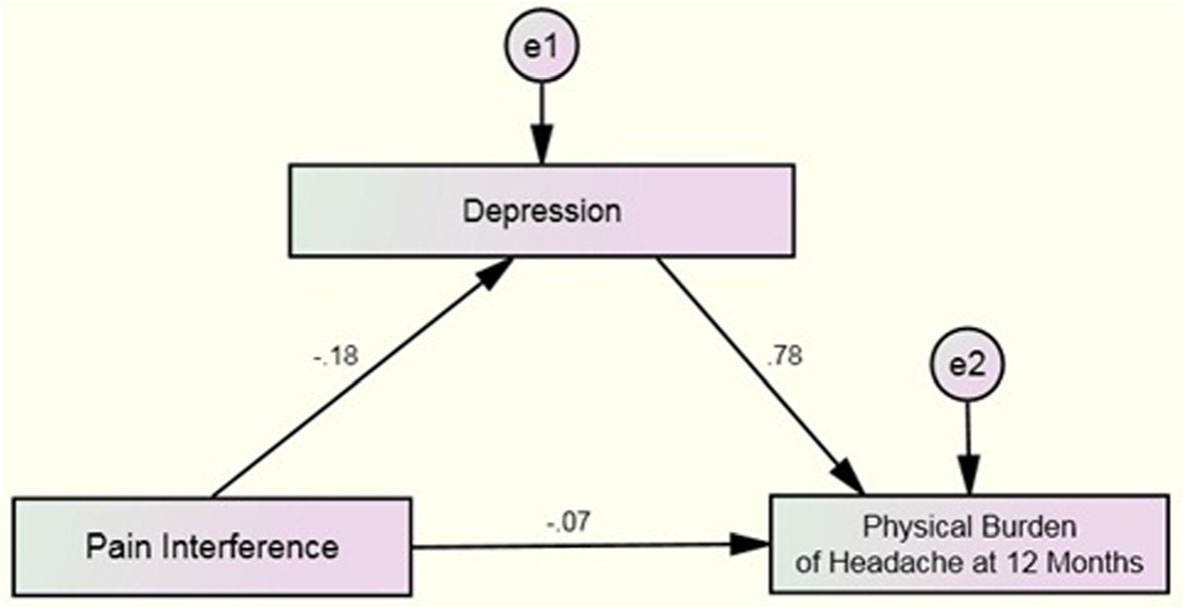
Mediation Analysis of Pain Interference on Physical Burden of Headache at one-year through Depression

Figure 1 summarizes the standardized effect of the first simple mediation model. First, the total effect from pain interference (bodily pain) on depression (HADS-D) was statistically significant (B = −0.18, *P* < .001). Second, the total direct effect from pain interference (bodily pain) on emotional burden of headache (HDI-E) at one-year was significant (B = −0.27, P < .001). Third, the total direct effect from depression (HADS-D) to the emotional burden of headache (HDI-E) at one-year was significant (B = 0.78, *P* < .001). Finally, the total indirect effect of pain interference on the emotional burden of headache (HDI-E) at one-year mediated through baseline depression (HADS-D) was also significant (B = −0.07, *P* = .04). To consider statistically significant the partial effect of mediation at this first model, Zobel test was significant (z = −2.52, *P* = .01) with a confidence level not including the value 0 (LLCI: -0.1546, ULCI: -0.0236).

**Fig. 2:**
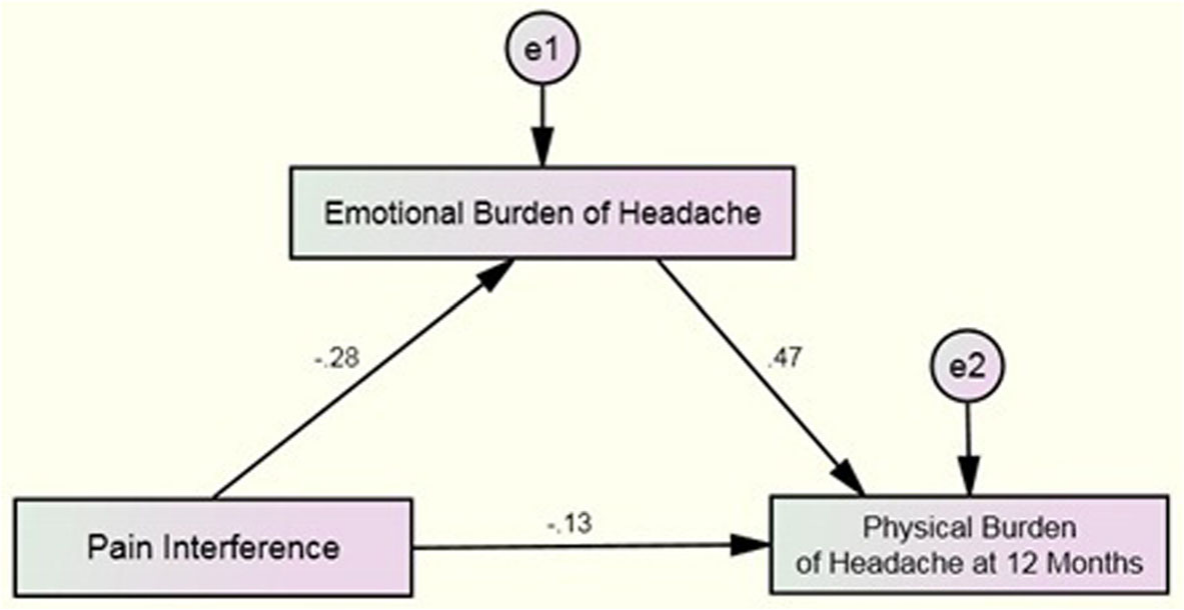
Mediation Analysis of Pain Interference on Physical Burden of Headache at one-year through Emotional Burden of Headache

The standardized effect of the second simple mediation model is shown in Fig. 2. First, the total effect from pain interference (bodily pain) on the emotional burden of headache (HDI-E) was statistically significant (B = −0.28, *P* < .001). Second, the total direct effect from pain interference (bodily pain) on the physical burden of headache (HDI-P) at one-year was also statistically significant (B = −0.19, *P* = .04). Third, the total direct effect from the baseline emotional burden of headache (HDI-E) on the physical burden of headache (HDI-P) at one-year was significant (B = 0.47, P < .001). Finally, the total indirect effect from pain interference (bodily pain) on the physical burden of headache at one-year mediated through the baseline emotional burden of headache (HDI-E) was also significant (B = −0.13, P = .04). Again, to consider the partial effect of mediation statistically significant at the second model, the Zobel test revealed that the model was significant (z = −3.74, *P* = .002) with a confidence level not including the value 0 (LLCI: -0.2200; ULCI: -0.0734).

## Discussion

This is the first longitudinal study investigating the variables associated with the burden of headache in individuals with CTTH. We observed that the emotional burden of headache was associated to baseline pain interference and depression whereas the physical burden of headache was associated to baseline emotional aspects of the burden, pain interference, and headache duration. Baseline depression and the emotional burden mediated the effects of pain interference with the emotional or physical burden at one-year follow-up, respectively.

The findings from this study show two important aspects. First, pain interference was longitudinally associated with both emotional and physical components of burden; and second, a relevant role of emotional aspects in patients with CTTH since depression and emotional component of burden indirectly mediated the effect of pain inference on the headache burden. Our results support an important association of pain interference with burden, which would agree with the conception that pain is a dimension associated to burden perception. In fact, the duration of the headache attack was also independently associated to the physical component of burden; supporting that not only the presence of pain, but also its duration, is relevant for burden perception. This may be related to the fact that pain interference refers to limitations on daily life activities due to the presence of pain and headache duration is related to the time with pain; therefore, this outcome reveals two different spheres of the pain spectrum.

We also found that the effects of pain interference on the emotional burden were mediated by depression, suggesting a relevant role of depression in patients with CTTH. It has been previously observed that depression has a marked impact on the burden in patients with chronic headaches since it increases the risk of feeling less understood by the family and friends as well as an increased risk of avoiding to tell other people about the headache [14]. In fact, it has been previously suggested that depression contributes to chronic pain via supra-spinal mechanisms and emotional modulation of pain [31]. Our results support this mechanism since depression mediated the effect of pain interference on the emotional burden of headache.

Similarly, the effect of pain interference on the physical burden was mediated by emotional aspects of burden, supporting a relevant role of emotional factors. It seems that emotional (stressful) factors are common precipitating factors of headache episodes in patients with TTH [32] since they may trigger hyperalgesic responses within the central nervous system [33]. Therefore, it is possible that emotional (stressful) factors can trigger more headache attacks inducing an increasing in the frequency of headaches, which would lead to worse pain interference and, therefore, higher self-perceived burden. In fact, the presence of mood disorders is more associated to the frequency of headaches rather than to headache diagnosis [34].

Uncertainty over biological mechanisms withstanding in these interactions, our results have clinical implications. Since emotional stress is the most common trigger for pain in subjects with TTH, proper management of emotional factors may be relevant for avoiding chronification and an increase of burden perception. In fact, emotional stress and depression represent two modifiable risk factors implicated in the progression from episodic to chronic headaches [35]; therefore, their management could also lead to better control of the headache burden. Our study found that depression and emotional aspects of burden were mediating factors in the association between pain interference and the burden of headache. Therefore, proper copying strategies for management of potential stressful emotional events and a reduction of depressive symptoms may be those factors associated with a reduction of the headache burden. Current findings would suggest that management of patients with CTTH should include therapeutic interventions targeted to decrease the emotional burden of headache (copying strategies or cognitive behavioral techniques) and to decrease depressive symptoms (i.e., psychological approaches) with the aim to decrease the burden of headache.

Although strengths of the current study include a large sample size, the inclusion of patients accordingly to the most updated diagnostic criteria, the use of diagnostic diaries and a longitudinal design, some limitations should be also recognized. First, we included patients with CTTH from a tertiary headache center; therefore, they may be not representative of the general population. Second, the impact of medication intake was not considered in the mediation models. Third, it should be noted that the HADS is a screening rather than diagnostic instrument for depressive symptoms with a tendency to underestimate its prevalence [36]. In fact, we noted that depression levels observed in our sample of CTTH patients were low; therefore, it is possible that the mediating effect between pain interference and the emotional burden of headache may be different in individuals experiencing higher levels of depression. Finally, we assessed the emotional or physical component of burden with the HDI. Previous studies assessing the burden of headache had used the HALT index which considers the days lost completely or partially because of headache in the preceding months and covers professional work, household activities or chores, and family, social or leisure activities. Although current and previous data [3] suggest that TTH should not be considered as a minimal form of headache due to its repercussions, current results should be considered in this context of headache burden, but not in the economic factor of headache.

## Conclusions

The current study found that pain interference and depression were longitudinally associated to the emotional burden of headache whereas the emotional headache burden, pain interference, and headache duration were longitudinally associated to the physical burden of headache in individuals with CTTH. Baseline depression and the emotional burden mediated the effects of pain interference with the emotional or physical burden at one-year follow-up, respectively. This study suggests that emotional factors play a relevant role in the association between pain interference and burden in patients with CTTH.

## Abbreviations

CTTH: Chronic tension type headache
HADS: Hospital Anxiety and Depression Scale
HDI: Headache Disability Inventory
ICHD3: International Classification of Headache Disorders, third edition
NPRS: Numerical Pain Rate Scale
PSQI: Pittsburgh Sleep Quality Index
SF-36: Health-related quality of life questionnaire
TTH: Tension type headache

## References

[1] Ferrante T, Manzoni GC, Russo M, Camarda C, Taga A, Veronesi L (2013). Prevalence of tension-type headache in adult general population: the PACE study and review of the literature. Neurol Sci.

[2] Dowson A (2015). The burden of headache: global and regional prevalence of headache and its impact. Int J Clin Pract Suppl.

[3] GBD 2016 Disease and Injury Incidence and Prevalence Collaborators (2017). Global, regional, and national incidence, prevalence, and years lived with disability for 328 diseases and injuries for 195 countries, 1990-2016: a systematic analysis for the global burden of disease study 2016. Lancet.

[4] Steiner TJ, Stovner LJ, Katsarava Z, Lainez JM, Lampl C, Lantéri-Minet M, Rastenyte D, Ruiz de la Torre E, Tassorelli C, Barré J, Andrée C (2014). The impact of headache in Europe: principal results of the Eurolight project. J Headache Pain.

[5] Raggi A, Leonardi M (2015). Burden and cost of neurological diseases: a European north-south comparison. Acta Neurol Scand.

[6] Linde M, Gustavsson A, Stovner LJ (2012). The cost of headache disorders in Europe: the Eurolight project. Eur J Neurol.

[7] Rastenytė D, Mickevičienė D, Stovner LJ, Thomas H, Andrée C, Steiner TJ (2017). Prevalence and burden of headache disorders in Lithuania and their public-health and policy implications: a population-based study within the Eurolight project. J Headache Pain.

[8] Zebenigus M, Tekle-Haimanot R, Worku DK, Thomas H, Steiner TJ (2017). The burden of headache disorders in Ethiopia: national estimates from a population-based door-to-door survey. J Headache Pain.

[9] Lampl C, Thomas H, Stovner LJ, Tassorelli C, Katsarava Z, Laínez JM, Lantéri-Minet M, Rastenyte D, Ruiz de la Torre E, Andrée C, Steiner TJ (2016). Interictal burden attributable to episodic headache: findings from the Eurolight project. J Headache Pain.

[10] Lampl C, Thomas H, Tassorelli C, Katsarava Z, Laínez JM, Lantéri-Minet M, Rastenyte D, Ruiz de la Torre E, Stovner LJ, Andrée C, Steiner TJ (2016). Headache, depression and anxiety: associations in the Eurolight project. J Headache Pain.

[11] Uhlig BL, Engstrøm M, Ødegård SS, Hagen KK, Sand T (2014). Headache and insomnia in population-based epidemiological studies. Cephalalgia.

[12] Rao AS, Scher AI, Vieira RV, Merikangas KR, Metti AL, Peterlin BL (2015). The impact of post-traumatic stress disorder on the burden of migraine: results from the national comorbidity survey-replication. Headache.

[13] Oh K, Cho SJ, Kim J, Chu MK (2014). Combination of anxiety and depression is associated with an increased headache frequency in migraineurs: a population-based study. BMC Neurol.

[14] Zebenholzer K, Lechner A, Broessner G, Lampl C, Luthringshausen G, Wuschitz A, Obmann SM, Berek K, Wöber C (2016). Impact of depression and anxiety on burden and management of episodic and chronic headaches - a cross-sectional multicentre study in eight Austrian headache centres. J Headache Pain.

[15] Palacios-Ceña M, Fernández-Muñoz JJ, Castaldo M, Wang K, Guerrero-Peral Á, Arendt-Nielsen L, Fernández-de-las-Peñas C (2017). The association of headache frequency with pain interference and the burden of disease is mediated by depression and sleep quality, but not anxiety, in chronic tension type headache. J Headache Pain.

[16] ICHD-III (2013). International classification of headache disorder: headache classification Subcommittee of the International Headache Society, 3nd edition. Cephalalgia.

[17] Jensen R, Tassorelli C, Rossi P, Allena M, Osipova V, Steiner T, Sandrini G, Olesen J, Nappi G, Basic Diagnostic Headache Diary Study Group (2011). A basic diagnostic headache headache diary (BDHD) is well accepted and useful in the diagnosis of headache. A multicentre European and Latin American study. Cephalalgia.

[18] Jensen MP, Turner JA, Romano JM, Fisher L (1999). Comparative reliability and validity of chronic pain intensity measures. Pain.

[19] Jacobson GP, Ramadan NM, Norris L, Newman CW (1994). The Henry ford hospital headache disability inventory. Neurology.

[20] Jacobson GP, Ramadan NM, Norris L, Newman CW (1995). Headache disability inventory (HDI): short-term test-retest reliability and spouse perceptions. Headache.

[21] Zigmond AS, Snaith RP (1983). The hospital anxiety and depression scale. Acta Psychiatr Scand.

[22] Herrmann-Lingen C, Buss U, Snaith R (2011) Hospital Anxiety and Depression Scale – Deutsche Version (HADS-D) Verlag Hans Huber, Bern

[23] Juang KD, Wang SJ, Lin CH, Fuh JL (1999). Use of the hospital anxiety and depression scale as a screening tool for patients with headache. Zhonghua Yi Xue Za Zhi (Taipei).

[24] Cole JC, Dubois D, Kosinski M (2007). Use of patient-reported sleep measures in clinical trials of pain treatment: a literature review and synthesis of current sleep measures and a conceptual model of sleep disturbance in pain. Clin Ther.

[25] Buysse DJ, Reynolds CF, Monk TH, Berman SR, Kupfer DJ (1989). The Pittsburgh sleep quality index: a new instrument for psychiatric practice and research. Psychiatry Res.

[26] Carpenter JS, Andrykowski M (1998). Psychometric evaluation of the Pittsburgh sleep quality index. J Psychosom Res.

[27] Ware JE, Sherbourne CD (1992). The MOS 36-item short-form health survey (SF-36). I. Conceptual framework and item selection. Med Care.

[28] McHorney CA, Ware JE, Raczek AE (1993). The MOS 36-item short-form health survey (SF-36): II. Psychometric and clinical tests of validity in measuring physical and mental health constructs. Med Care.

[29] Baron RM, Kenny DA (1986). The moderator-mediator variable distinction in social psychological research: conceptual, strategic and statistical considerations. J Personality Social Psychol.

[30] Hayes AF (2013). Introduction to mediation, moderation and conditional process analysis. A regression based approach.

[31] Terry EL, DelVentura JL, Bartley EJ, Vincent AL, Rhudy JL (2013). Emotional modulation of pain and spinal nociception in persons with major depressive disorder (MDD). Pain.

[32] Wang J, Huang Q, Li N, Tan G, Chen L, Zhou J (2013). Triggers of migraine and tension-type headache in China: a clinic-based survey. Eur J Neurol.

[33] Cathcart S, Petkov J, Winefield AH, Lushington K, Rolan P (2010). Central mechanisms of stress-induced headache. Cephalalgia.

[34] Zwart JA, Dyb G, Hagen K (2003). Depression and anxiety disorders associated with headache frequency: the Nord-Trøndelag health study. Eur J Neurol.

[35] Rains JC (2008). Chronic headache and potentially modifiable risk factors: screening and behavioral management of sleep disorders. Headache.

[36] Steel Z, Marnane C, Iranpour C, Chey T, Jackson JW, Patel V, Silove D (2014). The global prevalence of common mental disorders: a systematic review and meta-analysis 1980-2013. Int J Epidemiol.

